# A Review of Nanomaterials in Heavy-Oil Viscosity Reduction: The Transition from Thermal Recovery to Cold Recovery

**DOI:** 10.3390/nano16080452

**Published:** 2026-04-10

**Authors:** Zhen Tao, Borui Ji, Bauyrzhan Sarsenbekuly, Wanli Kang, Hongbin Yang, Wenwei Wu, Yuqin Tian, Sarsenbek Turtabayev, Jamilyam Ismailova, Ayazhan Beisenbayeva

**Affiliations:** 1 School of Energy and Petroleum Industry, Kazakh-British Technical University, Almaty 050000, Kazakhstan; tao349562421@126.com (Z.T.); jbr0108@163.com (B.J.); heakwww@163.com (W.W.); j.ismailova@kbtu.kz (J.I.); a.beisenbayeva@kbtu.kz (A.B.); 2 Liaoning Inspection, Examination & Certification Centre, Key Laboratory of Testing and Quality Control for Petroleum Products, State Administration for Market Regulation, Shenyang 110032, China; 3 School of Petroleum Engineering, China University of Petroleum (East China), Qingdao 266580, China; hongbinyang@upc.edu.cn; 4 Ningbo Fengcheng Advanced Energy Materials Research Institute Co., Ltd., Ningbo 315000, China; slcyytyq@sohu.com; 5 Faculty of Natural Science, Zhanibekov University, Shymkent 160012, Kazakhstan; sarsenbek.turtabayev@mail.ru

**Keywords:** heavy oil, viscosity reduction, nanoparticles (NPs), nano-assisted EOR, interfacial regulation

## Abstract

Heavy oil and extra-heavy oil represent mobility-limited petroleum resources because supramolecular associations of asphaltenes and resins, together with strong interfacial resistance, generate extremely high apparent viscosity. In recent years, nanotechnology has emerged as a promising approach for viscosity management and enhanced oil recovery (EOR). This review critically examines recent advances in nano-assisted viscosity reduction from a reservoir-operational perspective and organizes the literature into two field-relevant categories: metal-based and non-metal nano-systems. Metal-based nanoparticles (NPs) mainly promote catalytic aquathermolysis and related bond-cleavage and hydrogen-transfer reactions under hydrothermal conditions, enabling partial upgrading and persistent viscosity reduction during thermal recovery. In contrast, non-metal nano-systems—particularly silica- and graphene-oxide-derived materials—primarily operate through interfacial and structural regulation mechanisms at low or moderate temperatures. These effects include wettability alteration, interfacial-film stabilization, modification of asphaltene aggregation behavior, and the formation of dispersed-flow regimes such as Pickering-type emulsions that reduce apparent flow resistance in multiphase systems. Beyond summarizing nanomaterial types, this review emphasizes reservoir-scale considerations governing field applicability, including brine stability, NPs transport and retention in porous media, and formulation compatibility. Comparative analysis highlights the distinct operational windows of thermal catalytic nano-systems and cold-production nano-systems, providing a reservoir-oriented framework for designing nano-assisted viscosity-reduction technologies.

## 1. Introduction

With the gradual decline of conventional light oil production, heavy and extra-heavy oil resources have become an increasingly important component of future petroleum supply. However, unlike conventional crude oils, the development of heavy oil is primarily constrained by poor mobility rather than resource availability. The extremely high viscosity of heavy oil leads to strong flow resistance in porous media, large pressure gradients, and low displacement efficiency during production. As a result, improving heavy-oil mobility has become a central objective in reservoir development and production engineering. Under the current context of energy transition and increasing environmental constraints, it is necessary to re-examine viscosity-reduction strategies and their practical applicability in heavy-oil production systems.

### 1.1. Challenges in Heavy Oil Development in the Context of Energy Transition

Although clean and low-carbon energy sources (e.g., solar, wind, geothermal, hydropower, biomass, and nuclear) have expanded rapidly in recent years, they remain insufficient to replace fossil fuels at the global scale. Data from the International Energy Agency (IEA) and the OPEC World Oil Outlook 2050 indicate that fossil fuels will remain an important component of the global energy system for the foreseeable future [1,2,3].

As the production of conventional light crude oil enters a phase of natural maturity and decline, the energy industry has increasingly shifted its strategic focus toward unconventional energy resources to ensure long-term supply stability [1,3]. Among these resources, heavy oil and extra-heavy oils are particularly abundant. However, the primary challenge in heavy-oil development is poor mobility rather than resource availability, because the extremely high viscosity generates large start-up pressures and strong flow resistance in porous media, often rendering conventional waterflooding ineffective. Although thermal recovery can alleviate mobility limitations, it is commonly associated with high energy consumption, significant water demand, and increased carbon intensity. From an engineering standpoint, a more practical route is to treat viscosity reduction and flow improvement as the core of chemical-system design, aiming to improve heavy-oil mobility under low-energy and low-shear conditions [4].

Current estimates indicate that heavy oil constitutes a substantial portion of the world’s remaining petroleum resources [5,6,7,8,9,10,11,12]. Major heavy oil accumulations are distributed across regions such as the Orinoco Belt in Venezuela, the Athabasca oil sands in Canada, western and northern China, the UK Continental Shelf, the Llanos Basin in Colombia, and the Duri Basin in Indonesia [13,14]. In China, heavy oil is commonly reported to account for ~25% of total oil and gas resources (in-place), exceeding 20 billion tons, with >4 billion tons considered technically recoverable [15]. These resources are primarily concentrated in the Liaohe, Bohai, Shengli, Xinjiang, Tarim, and Tahe oilfields [16]. Given their strategic importance, the efficient development of heavy and extra-heavy oil resources is closely tied to national energy security [17].

### 1.2. Viscosity Thickening Mechanisms of Heavy Oil

Unlike conventional light oils, many heavy oils exhibit non-Newtonian rheological characteristics; they are, in fact, complex colloidal systems mainly composed of four components: saturates, aromatics, resins, and asphaltenes, etc., which are commonly known as the SARA components [18]. Heavy crude oil is also typically rich in heteroatoms (e.g., N, O, and S, etc.) and coordinated metal species such as Ni and V; consequently, the high viscosity of heavy oil primarily stems from the interactions among resins, asphaltenes, and these heteroatoms [19,20,21].

**Figure 1:**
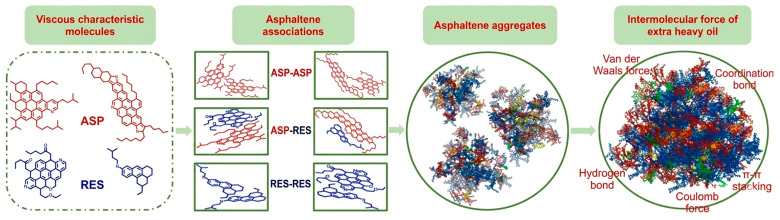
The mechanism of high-viscosity extra-heavy oil formation. Adapted from Ref. [22] (ASP: asphaltene; RES: resin).

Asphaltenes and resins can self-associate into supramolecular structures through π–π stacking, hydrogen bonding, electrostatic interactions, and metal coordination (Figure 1) [22,23,24,25]. Figure 1 shows the major interactions and formation process of highly viscous extra-heavy oil. These associations form a weak three-dimensional network that governs the rheological behavior of heavy oil, leading to non-Newtonian characteristics such as shear-thinning, viscoelasticity, and, in some cases, yield stress.

When asphaltenes are abundant and resins are insufficient to stabilize them in a dispersed state, they associate into larger clusters. These clusters can bridge and connect, forming a continuous viscoelastic structure and markedly increasing the flow resistance [26]. This network markedly increases flow resistance, resulting in poor fluidity and hindering heavy-oil production and transport [6,27].

Therefore, viscosity-reduction strategies fundamentally rely on disrupting or modifying these association networks. From a field perspective, the relevant viscosity window differs between recovery modes: thermal processes can target extremely high viscosities (up to ~10^6^ mPa·s), whereas cold-production-oriented systems typically operate in the range of ~10^3^–10^4^ mPa·s, where mobility improvement is mainly achieved through interfacial and structural regulation rather than irreversible upgrading.

### 1.3. Evolution of Viscosity-Reduction Technologies

Given the complex composition and extremely high viscosity of heavy oil, its extraction continues to face significant technical challenges [28]. Heavy oil is generally characterized by high viscosity (typically > 100 mPa·s) and high density (API gravity < 20°). In extreme cases, such as extra-heavy oils, viscosity may exceed 1,000,000 mPa·s. High viscosity results in an unfavorable mobility ratio, leading to viscous fingering, early water breakthrough, and reduced oil recovery. Therefore, viscosity reduction and mobility improvement are prerequisites for heavy-oil production, transportation, and utilization [29].

**Figure 2:**
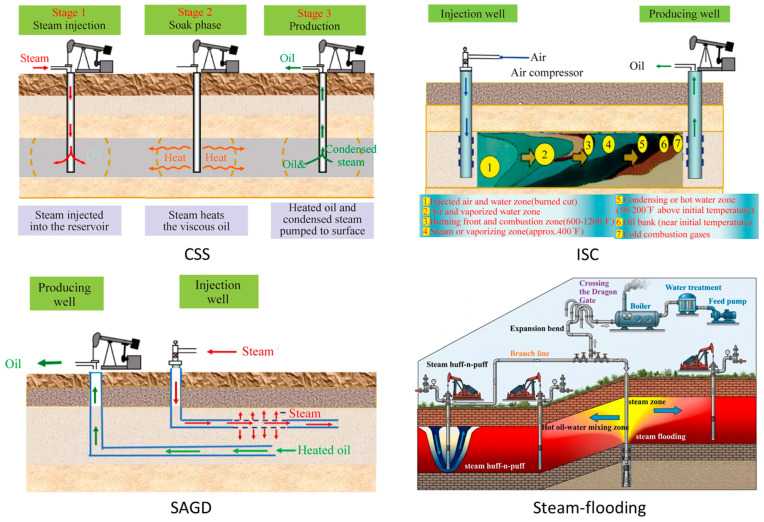
The thermal recovery technologies. Adapted from Refs. [7,32].

In practice, heavy-oil viscosity-reduction technologies are broadly classified into two categories: thermal recovery and cold recovery [30]. Thermal recovery is widely applied because heavy oil is highly sensitive to temperature [29]. These methods reduce viscosity by heating the reservoir, thereby improving recovery efficiency [31]. Thermal technologies have been widely developed, including cyclic steam stimulation (CSS), steam flooding, steam-assisted gravity drainage (SAGD), in situ combustion (ISC), and thermochemical combined flooding (Figure 2) [7,30,32,33,34,35,36,37,38]. These approaches typically rely on high-temperature steam injection to substantially reduce viscosity [39].

However, these methods also present significant operational drawbacks. First, thermal recovery requires large volumes of high-quality water and substantial energy input, resulting in high capital and operating costs [40]. Second, steam generation and fuel combustion during thermal operations can produce substantial greenhouse gas (GHG) emissions [41]. Third, significant heat loss to overburden and underlying formations reduces thermal efficiency, particularly in thin, deep, or heterogeneous reservoirs [42]. In addition, irreversible thermal cracking and coke formation under high-temperature conditions may impair porous media and reduce permeability [43]. Overall, the primary limitations of thermal recovery include high energy consumption, associated carbon emissions, and constraints related to reservoir adaptability and economic feasibility [44,45]. Consequently, thermal recovery often shows limited applicability in moderate-viscosity heavy-oil reservoirs.

Consequently, to address the limitations of traditional thermal recovery, researchers have proposed various non-thermal techniques to reduce the viscosity of heavy oil [46], including dilution with light hydrocarbons (e.g., Naphtha, condensate, CO_2_, hydrocarbon gases, etc.) [47,48], chemical viscosity reduction methods (such as surfactants, dispersant viscosity reducer) [46,49,50]. However, these methods usually show insurmountable limitations in practical applications [51].

In recent decades, various alternative technologies have been explored to overcome the limitations of conventional viscosity-reduction approaches, including catalytic upgrading, microbial processes, ultrasonic methods, and nanoparticle-assisted techniques [7,27,52,53,54,55,56]. Among these approaches, nanomaterials have attracted increasing attention because their nanoscale size, large surface area, and tunable surface chemistry allow them to interact with crude-oil components and reservoir interfaces at the molecular level [57]. As a result, nanotechnology has emerged as a promising direction for improving heavy-oil mobility and enhancing oil recovery.

**Figure 3:**
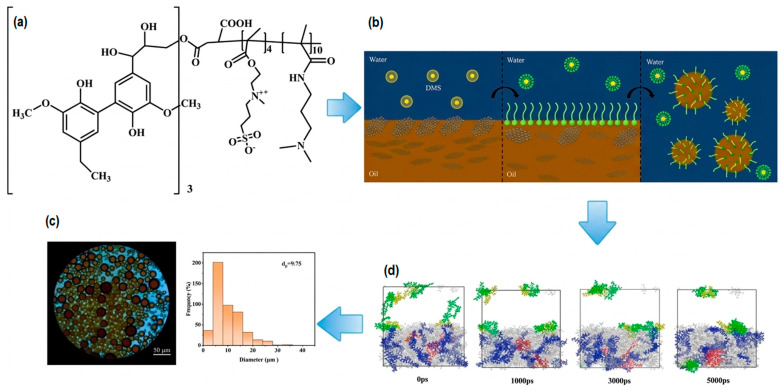
Schematic illustration of lignin-based amphoteric surfactant systems and their role in heavy-oil viscosity reduction and mobility improvement. Adapted from Ref. [46]. (**a**) Molecular structure of lignin-based amphoteric surfactants; (**b**) interfacial mechanism of viscosity reduction, including adsorption at the oil–water interface and stabilization of dispersed oil droplets (Pickering-type behavior); (**c**) optical micrograph and corresponding particle size distribution of heavy-oil emulsions after surfactant treatment; (**d**) molecular dynamics simulation showing the time-dependent evolution of asphaltene aggregation and dispersion (0–5000 ps), indicating disruption of supramolecular networks. These coupled molecular and interfacial processes can promote dispersed-flow regimes in porous media, thereby reducing apparent flow resistance and improving heavy-oil mobility during displacement.

Among the proposed strategies, emulsification-based nano-chemical flooding has received particular attention [46]. In such systems, NPs and amphiphilic agents can adsorb at the oil–water interface, stabilize dispersed oil droplets, and modify interfacial films, thereby promoting water-continuous flow regimes. As illustrated in Figure 3, these coupled molecular and interfacial processes can disrupt asphaltene aggregation, stabilize dispersed structures, and reduce apparent flow resistance, ultimately improving heavy-oil mobility during displacement.

The studies summarized in Table 1 reveal that nano-assisted viscosity reduction in heavy oil has been investigated through several distinct research directions. Early studies on metal and metal-oxide NPs mainly focused on catalytic upgrading mechanisms under hydrothermal conditions, where NPs promote aquathermolysis reactions and facilitate the cleavage of heavy hydrocarbon structures. In contrast, a growing body of literature on silica and carbon-based nanomaterials highlights mechanisms related to interfacial adsorption, stabilization of dispersed oil droplets, and modification of asphaltene aggregation behavior. These observations suggest that nano-assisted viscosity reduction can broadly operate through two different mechanisms depending on reservoir conditions and system design. Finally, despite promising laboratory results, many nano-systems still face practical constraints related to NPs transport, stability in brine, and injectivity under reservoir conditions.

Recent studies increasingly suggest that nano-assisted viscosity reduction can operate through multiple mechanisms depending on reservoir conditions and operating strategies. Early work mainly focused on metal and metal-oxide NPs for in situ upgrading under thermal conditions. However, more recent studies have increasingly explored non-metallic nanomaterials (e.g., silica or carbon-based systems) that can regulate interfacial properties and improve heavy-oil mobility under low-temperature or cold-production conditions.

Unlike many previous reviews that primarily summarize NPs categories or laboratory viscosity-reduction results, the present work aims to provide a more analytical synthesis of the literature from a field-operational perspective. Therefore, from a reservoir-engineering perspective, nano-assisted viscosity reduction may operate through fundamentally different mechanisms depending on whether the system is applied under thermal recovery conditions or cold-production environments. Specifically, this review distinguishes between two fundamentally different mechanisms of nano-assisted viscosity reduction based on reservoir operating conditions: (i) thermal-bound catalytic upgrading, which requires sustained hydrothermal conditions to activate aquathermolysis reactions; and (ii) interfacial–structural regulation, which improves heavy-oil mobility under cold-production conditions through wettability alteration, dispersion stabilization, and modification of asphaltene association networks.

By organizing the literature around these mechanisms and linking them to reservoir-scale constraints such as brine stability, NPs transport, retention, and injectivity, this review provides a framework that connects laboratory observations with practical field considerations. In particular, non-metallic nano-systems may offer promising opportunities for improving heavy-oil mobility under cold-production conditions where thermal recovery is less economically attractive.

To maintain conceptual clarity in the following sections, several related terms used in this review are defined below. “Nanomaterials” refers to the NPs materials themselves (e.g., silica NPs, graphene oxide, or metal oxides) considered as functional components. “Nanofluids” refers to dispersed systems in which NPs are suspended in a carrier fluid and injected into porous media. The broader term “nano-systems” is used to describe integrated formulations in which nanomaterials are combined with other chemical agents (e.g., surfactants or polymers) to achieve viscosity reduction and mobility control under reservoir conditions.

Three related functional terms are also distinguished in this review. “Viscosity reduction” refers to a decrease in the intrinsic viscosity of the oil phase or to values reported as such in the literature. “Mobility improvement” refers to enhanced transport behavior in porous media resulting from mechanisms such as wettability alteration, interfacial regulation, or dispersed-flow formation, even when the intrinsic oil viscosity changes only moderately. “Flow improvement” is used as a broader engineering term describing reductions in flow resistance, pressure drop, or displacement constraints at the system level.

## 2. Metal-Based Nanomaterials for Heavy Oil Thermal Recovery

Metal-based nanomaterials are among the earliest and most extensively investigated nano-catalysts for heavy-oil viscosity reduction [65,66]. Under hydrothermal conditions, metal or metal-oxide NPs can catalyze aqua-thermolysis and related bond-cleavage/hydrogen-transfer reactions, facilitating in situ upgrading of heavy fractions (resins/asphaltenes) and thereby reducing viscosity [67,68]. Because heavy-oil viscosity decreases strongly with temperature, steam-based thermal recovery processes (e.g., CSS, steam flooding, and SAGD) are widely applied in practice [69]. The incorporation of metal-based NPs into such processes aims to enhance heat utilization by coupling thermal thinning with catalytic upgrading, thereby transforming temporary viscosity reduction into more persistent chemical modification [65,67,68].

### 2.1. Catalytic Aquathermolysis Mechanism

**Figure 4:**
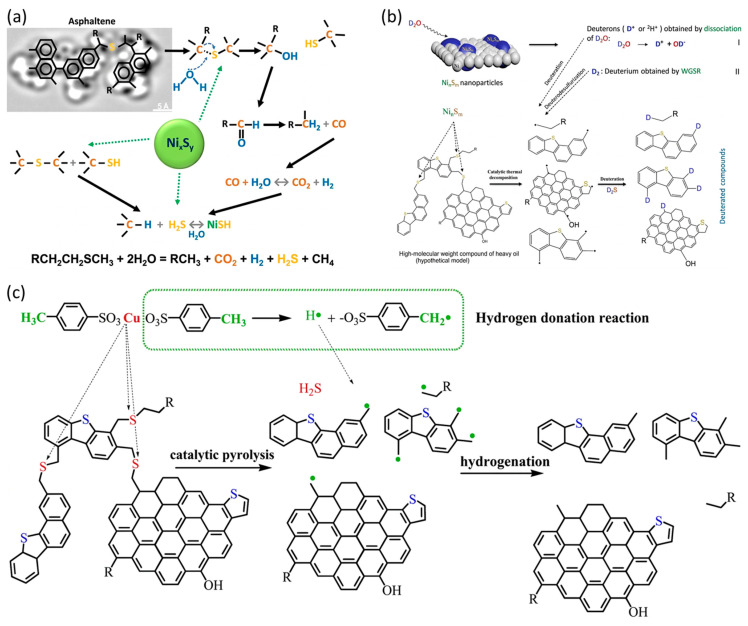
Schematic diagram illustrating the thermal cracking of heavy oil using metal NPs. Adapted from Ref. [71]. (**a**) The schematics of main chemical reactions during the aquathermolysis process in the presence of nickel sulfide catalyst; (**b**) deuterium generation in the presence of NinSm NPs during thermal cracking of heavy oil molecules; (**c**) the destruction mechanism of C-S bonds.

The dominant viscosity-reduction pathway associated with metal-based nanomaterials is catalytic aqua thermolysis, i.e., hydrothermal upgrading promoted by dispersed metal/metal-oxide catalysts under steam/hot-water conditions (Figure 4) [70,71]. Under high-temperature and high-pressure conditions, metal NPs catalyze reactions between water and heavy oil components, which preferentially cleave heteroatom-containing linkages (e.g., C-S, C-N, and C-O bonds) and can partially crack alkyl side chains attached to aromatic cores [71,72].

These reactions decrease the average molecular weight of heavy fractions and weaken intermolecular association networks among asphaltenes and resins, as reflected by SARA redistribution and viscosity reduction after catalytic treatment [67,73]. In addition, many metal-based systems promote hydrogen-transfer pathways, often coupled with reactions such as the water–gas shift, generating reactive hydrogen species in situ [74,75]. These species stabilize cracking-derived radicals and suppress secondary condensation or coke formation.

As a result, catalytic aquathermolysis leads to irreversible compositional upgrading, providing more persistent viscosity reduction than purely physical approaches [67,70,73]. However, its effectiveness is strongly governed by temperature and water/steam availability, which control reaction kinetics and catalyst activation [70,71].

### 2.2. Representative Metal-Based Nanomaterials

A variety of metal-based nanomaterials have been explored for catalytic aquathermolysis, including transition metals (e.g., Ni, Co, Fe), metal oxides (e.g., Fe_3_O_4_, Fe_2_O_3_, Al_2_O_3_, TiO_2_, CeO_2_), and bimetallic or supported systems designed for hydrothermal upgrading [66,68,76].

**Figure 5:**
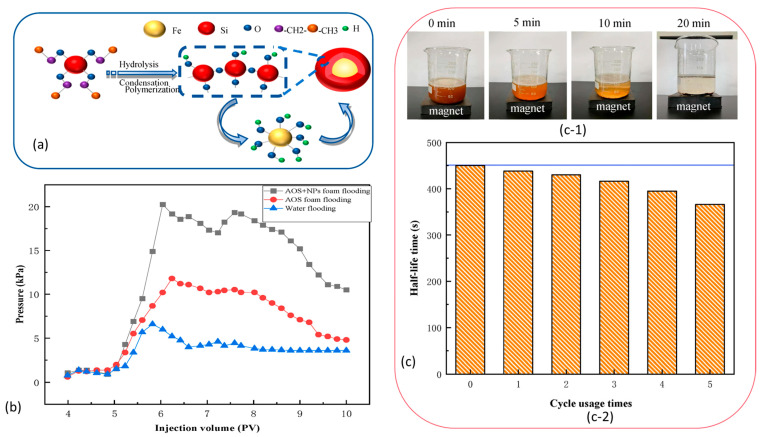
Surface-functionalized Fe_3_O_4_ NPs for EOR. Adapted from Ref. [78]. (**a**) Schematic representation of the synthesis mechanism of Fe_3_O_4_@SiO_2_ NPs; (**b**) the pressure of flooding experiments of Fe_3_O_4_@SiO_2_ NPs; (**c**) evaluation of recyclability of NPs: (**c-1**) the responsiveness of NPs to a magnet; (**c-2**) the drainage half-life time changes with the recycle number of NPs.

Among these, iron-based NPs (especially Fe_3_O_4_/Fe_2_O_3_) are widely studied due to their low cost and magnetic properties, which facilitate dispersion control and potential recovery (Figure 5) [77,78]. Surface functionalization can further improve colloidal stability and catalytic performance under hydrothermal conditions [77].

Nickel-based catalysts (e.g., NiO, Ni_x_O_x_) are often associated with enhanced heteroatom removal and hydrogen redistribution, making them particularly relevant for sulfur-rich heavy oils [76]. To improve activity and stability, bimetallic systems (e.g., Ni–Fe, MFe_2_O_4_) have been developed, leveraging synergistic redox and bond-cleavage pathways, although at the cost of increased system complexity [66,72].

In addition to conventional steam-based processes, metal-based NPs have also been combined with microwave or electromagnetic heating, where they function as both catalytic centers and localized heat sources [79,80].

Despite these diverse designs, reported performance varies widely. Under hydrothermal conditions (typically 180–300 °C), viscosity reductions generally range from ~30% to >90%, depending on catalyst composition, operating conditions, and crude-oil properties [67,70,76]. Fe- and Ni-based systems commonly induce partial upgrading, with reported decreases in asphaltene content (~5–20%) accompanied by increases in lighter fractions [71,73].

More importantly, these variations reveal consistent trends across different nano-systems. Higher temperatures and sufficient water availability generally enhance catalytic efficiency, while catalyst composition influences selectivity toward bond cleavage and hydrogen-transfer pathways. At the same time, crude-oil composition—particularly asphaltene content and heteroatom distribution—plays a critical role in determining the extent of upgrading.

To facilitate systematic comparison, representative catalytic aquathermolysis studies are summarized in Table 2, where key parameters including catalyst type, operating temperature, concentration, and viscosity-reduction performance are compiled.

As shown in Table 2, iron- and nickel-based systems typically achieve moderate-to-high viscosity reduction (∼40–90%) under hydrothermal conditions, whereas supported or rare-earth-based catalysts (e.g., CeO_2_) often exhibit more moderate performance. These differences are closely associated with variations in reaction conditions and catalytic pathways rather than intrinsic material properties alone.

Overall, the comparison indicates that catalytic performance is governed by coupled system-level factors—including catalyst composition, hydrothermal conditions, and crude-oil characteristics—highlighting the importance of evaluating nanomaterials within a consistent experimental and operational framework.

### 2.3. Advantages of Metal-Based Nano-Systems

Metal-based nanomaterials offer several advantages when the target mechanism is reaction-driven upgrading. Most importantly, they enable irreversible viscosity reduction by catalyzing aquathermolysis reactions that convert heavy fractions into lighter components [65,66,68,70].

In addition, their catalytic function can be directly integrated into existing steam-based recovery processes, utilizing the hydrothermal environment to enhance upgrading reactions and improve oil mobility [65,66,68].

Furthermore, the mechanistic understanding of catalytic aquathermolysis has been progressively strengthened, with recent studies incorporating kinetic descriptions and coupled reaction–transport modeling to better describe upgrading behavior under reservoir-relevant conditions [70,81,82].

These features make metal-based nano-systems most suitable for reservoirs where thermal recovery is already feasible and sufficient heat input can be maintained [64,66].

### 2.4. Limitations and Applicability Constraints

A fundamental limitation of metal-based nanomaterials is their strong dependence on elevated temperatures. Without sustained hydrothermal conditions, catalytic aquathermolysis proceeds too slowly to achieve meaningful viscosity reduction, making these systems unsuitable for cold production [65,66,70].

More importantly, their applicability is primarily constrained by reservoir conditions rather than material properties alone. In relatively low-temperature reservoirs, or in cases where heavy-oil viscosity falls within a moderate range (∼300–10,000 mPa·s), the incremental benefit of catalytic upgrading is often limited [65,66,70]. Under such conditions, mobility improvement can typically be achieved more efficiently through non-thermal approaches, without the need for energy-intensive thermal operations [65,66].

Furthermore, challenges related to NPs transport and retention remain significant. Under reservoir conditions, NPs may adsorb onto mineral surfaces, aggregate, or become trapped in pore throats, potentially causing injectivity decline and limited propagation [83,84].

In addition, not all reservoirs are suitable for steam-based recovery. In formations prone to steam channeling, rapid heat loss, or poor thermal confinement, the efficiency of heat utilization is significantly reduced, leading to high operational costs [85,86]. In such cases, the hydrothermal conditions required for catalytic activation cannot be effectively maintained, limiting the practical advantage of metal-based nano-systems [65,70].

From a broader perspective, metal-based nano-systems remain inherently coupled to energy- and water-intensive thermal recovery processes and therefore do not fundamentally alter the development paradigm of heavy-oil production [85,86].

Overall, these constraints indicate that metal-based nanomaterials are most applicable in reservoirs where stable and efficient thermal operations can be sustained. This reservoir-dependent applicability has driven increasing interest in non-thermal nano-assisted strategies, in which viscosity reduction is achieved through interfacial and structural regulation rather than catalytic upgrading [87,88].

## 3. Non-Metal Nanomaterials for Heavy-Oil Viscosity Reduction

Non-metal nanomaterials have emerged as an alternative to metal-based systems for heavy-oil viscosity reduction, particularly under conditions where thermal recovery is limited or impractical [87,89]. Unlike catalytic aquathermolysis, these systems operate primarily through interfacial and structural regulation at low-to-moderate temperatures rather than high-temperature upgrading reactions [88]. Their effects are best understood as coupled physicochemical processes, involving wettability alteration, interfacial tension reduction, stabilization of dispersed oil–water systems, and modification of asphaltene aggregation behavior [46,57,60,89,90]. These interactions collectively weaken oil–rock adhesion, disrupt asphaltene associations, and promote dispersed-flow regimes, thereby reducing apparent flow resistance and enhancing mobility. Their performance is strongly dependent on reservoir conditions, including salinity, temperature, and crude-oil composition, and varies across different nano-systems with distinct functional roles and operational constraints. Accordingly, the following sections examine representative non-metal nanomaterials—including silica-based NPs, graphene-oxide-derived systems, and functionalized nano-systems—with emphasis on their mechanisms, performance characteristics, and applicability under reservoir-relevant conditions.

### 3.1. Silica-Based Nano-Systems: Interfacial Film Engineering and Wettability Regulation

**Figure 6:**
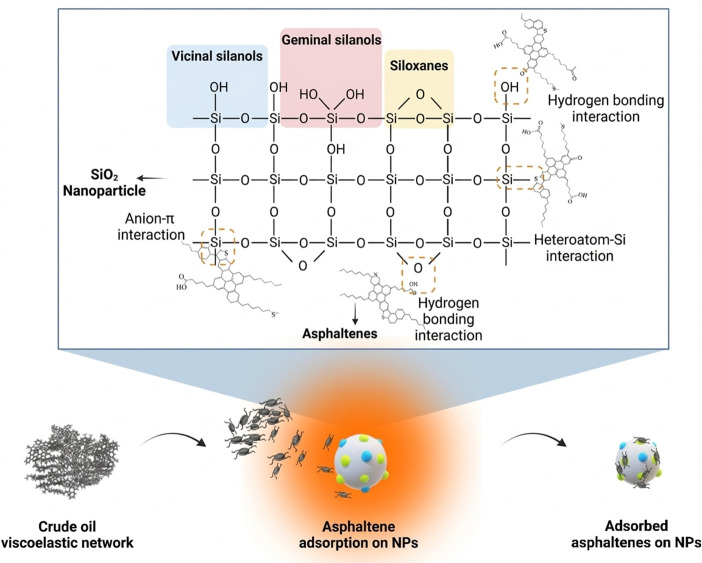
Graphical illustration of the proposed mechanisms for crude oil viscoelastic network disruption by asphaltene adsorption on SiO_2_ NPs. Adapted from Ref. [87].

Silica NPs are among the most extensively investigated non-metal nanomaterials for heavy-oil viscosity reduction and enhanced oil recovery (EOR). To better interpret their reported effects, the experimental framework commonly used to evaluate nanofluid-assisted viscosity reduction is illustrated in Figure 6. In most studies, silica NPs are first dispersed in aqueous media to form stabilized nanofluids and then introduced into heavy-oil systems under non-thermal conditions. Their performance is commonly evaluated through viscosity measurements, emulsion behavior, and transport characteristics in porous media [87,89].

Silica-based nano-systems are generally considered to interact with the supramolecular organization of heavy components, particularly the association networks formed by asphaltenes and resins. However, in many studies, the proposed disruption or modification of these networks is not directly observed at the molecular scale but is instead inferred from macroscopic changes in viscosity, rheology, and flow behavior [91,92,93]. Therefore, their main role is more reasonably interpreted as interfacial and structural regulation rather than direct cleavage or degradation of asphaltene molecular frameworks [94].

At the pore scale, viscosity reduction in silica-based systems is usually associated with several coupled effects, including adsorption-mediated modification of asphaltene aggregation, interfacial film regulation, and wettability alteration that improves oil mobility in porous media [95]. Hydrophilic silica NPs can adsorb onto mineral surfaces and thereby weaken oil adhesion at the oil–solid interface [96]. This process promotes oil-film detachment and shifts wettability toward more water-wet conditions, which improves displacement efficiency and indirectly reduces apparent viscosity during porous-medium flow.

When the particle surface is partially hydrophobic or surface-modified, silica NPs tend to accumulate at the oil–water interface and stabilize oil-in-water emulsions through Pickering-type mechanisms [97]. In addition, the interfacial co-adsorption of silica NPs with indigenous surface-active species such as asphaltenes can further stabilize interfacial films and modify droplet interactions [98]. From a rheological perspective, these processes transform the transport mode from continuous high-viscosity oil flow into the migration of dispersed oil droplets within a water-continuous phase. Under such conditions, it is important to distinguish intrinsic viscosity reduction in the oil phase from apparent viscosity reduction in the flowing system [99].

**Figure 7:**
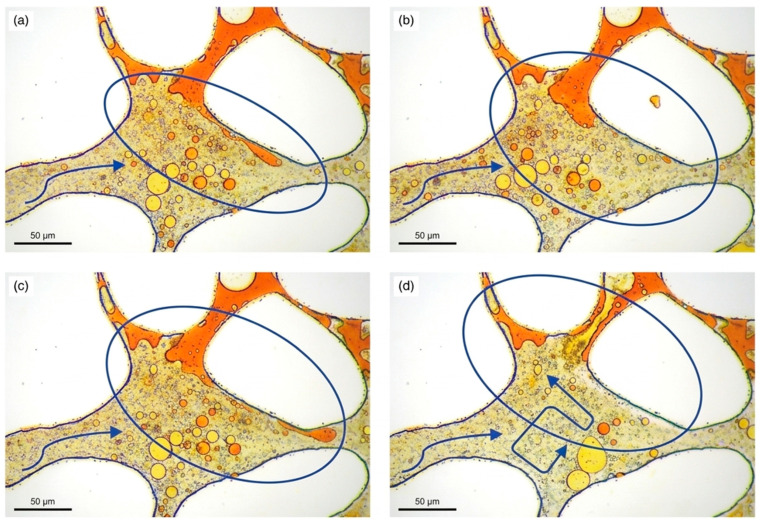
Schematic diagram of the microscopic visualization of the viscosity reduction in heavy oil by amine-modified silica NPs Pickering emulsions. Adapted from Ref. [100]. (**a**) Trapped oil clusters after waterflooding; (**b**) interfacial instability and droplet generation; (**c**) formed mobile O/W emulsions; (**d**) O/W emulsion migration through pore networks.

Accordingly, viscosity measurements alone do not provide direct evidence of molecular-level modification in heavy oil. Instead, the observed viscosity reduction should be interpreted mainly as a consequence of interfacial and structural regulation associated with dispersed-flow behavior. Under reservoir-relevant conditions, effective silica loadings are often reported in the range of approximately 100–5000 ppm, depending on salinity, oil–water ratio, and shear history (Figure 7) [100]. Overall, the reviewed studies indicate that silica-based nano-systems reduce flow resistance primarily through interfacial film engineering, wettability regulation, and dispersed-flow formation, which explains their relevance to cold heavy-oil production.

### 3.2. Carbon-Based Nano-Systems: Structural Interference with Asphaltene-Rich Networks

Carbon-based nanomaterials provide a viscosity-reduction pathway that is closely related to their ability to interact directly with asphaltene- and resin-rich domains in heavy oil. Among these materials, graphene oxide (GO) has received particular attention because of its lamellar two-dimensional structure, large specific surface area, and abundant oxygen-containing functional groups, which provide multiple tunable interaction sites with polar aromatics and mineral surfaces [92,101,102,103,104,105]. These characteristics enable GO nanosheets to participate simultaneously in structural interactions with heavy components and in interfacial processes that influence multiphase flow behavior.

Compared with zero-dimensional NPs, GO nanosheets possess large lateral dimensions and atomic-scale thickness, which allow them to insert into asphaltene nanoaggregates and interfere with π–π stacking between aromatic cores. This structural interference weakens the connectivity of the supramolecular network and renders the aggregate structure less stable under shear deformation [102,106,107]. As a result, several experimental studies have reported viscosity reduction accompanied by rheological changes such as decreased elastic response and weakened network coherence, which are consistent with a “structural disturbance” mechanism involving disruption of asphaltene aggregation rather than thermal cracking or permanent chemical transformation [102,106,107].

**Figure 8:**
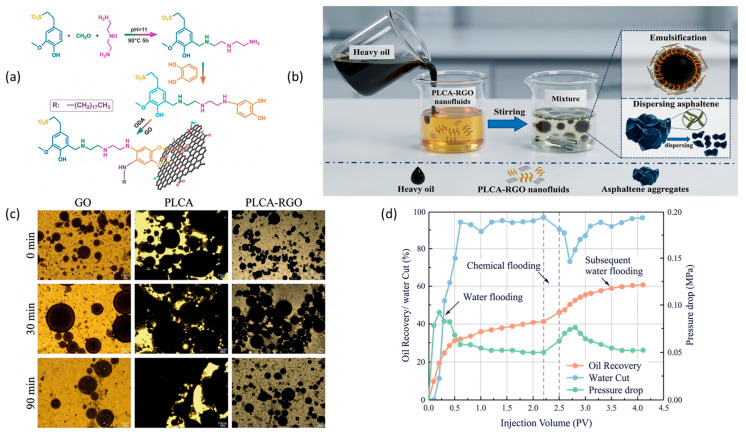
Novel poly(catecholamine)-modified GO nanosheets show a significant effect in improving the recovery rate of conventional heavy oil. Adapted from Ref. [92]. (**a**) Possible polymerization mechanism of the PLCA-RGO composite; (**b**) viscosity reduction mechanism diagram of the PLCA-RGO; (**c**) microscopic images of the emulsions formed by GO, PLCA, and PLCA-RGO at different times; (**d**) cumulative oil recovery, water cut, and pressure drop as a function of injected pore volume.

The structural-interference mechanism is schematically illustrated in Figure 8 using a poly(catecholamine)-modified GO system. In this example, GO nanosheets interact with heavy-oil components and assist in the formation of dispersed emulsified structures, ultimately contributing to improved oil mobility and measurable recovery enhancement during displacement experiments [92]. These observations illustrate how carbon-based nanosheets can simultaneously influence both microstructural organization and multiphase flow behavior.

GO also exhibits a strong tendency to associate with asphaltenes through noncovalent interactions such as π–π stacking and hydrogen bonding [92,102,103,104,108]. In many viscosity-reduction formulations, this association functions primarily as a localization mechanism that allows nanosheets to access asphaltene-enriched domains rather than as an adsorption-dominated removal process. Because these interactions are reversible and condition-dependent (for example, influenced by salinity and pH), the resulting structural interference may occur without causing severe or irreversible NPs retention within porous media.

**Figure 9:**
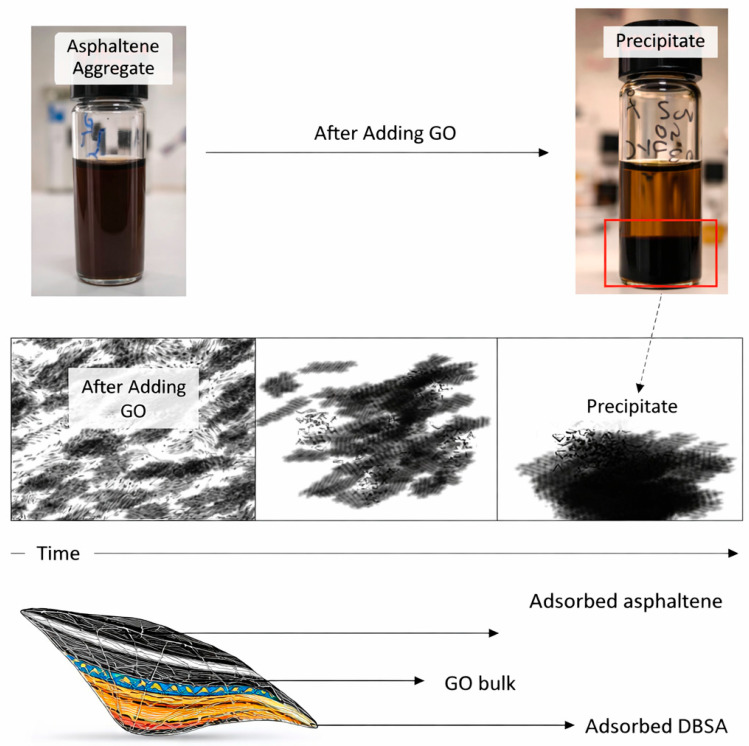
From left to right: the stable asphaltene dispersed in the micelle precursor, after introduction of GO to the asphaltene aggregate solution. Asphaltene aggregates are stable, while the sample in which GO is added has precipitated. In addition, an illustration of the precipitated structure and the mechanism of destabilization and precipitation. Adapted from Ref. [102].

Direct experimental evidence for the destabilization of asphaltene aggregates in the presence of GO is shown in Figure 9. After the introduction of GO nanosheets, previously stable asphaltene dispersions can become destabilized, leading to precipitation or restructuring of aggregates [102]. These observations support the interpretation that GO disrupts the supramolecular organization of heavy components, thereby weakening the viscoelastic network responsible for high viscosity.

At the formulation level, GO nanosheets are typically effective at relatively low concentrations, commonly in the range of approximately 10–500 ppm and occasionally up to about 1000 ppm when stabilized using surfactant or polymer functionalization [92,103,108]. In addition to structural interactions with asphaltene-rich domains, surface-modified GO systems can also regulate oil–water interfacial properties, including contact angle and interfacial tension (IFT) [104]. Such interfacial regulation can stabilize dispersed oil droplets and improve sweep efficiency during porous-medium displacement.

**Figure 10:**
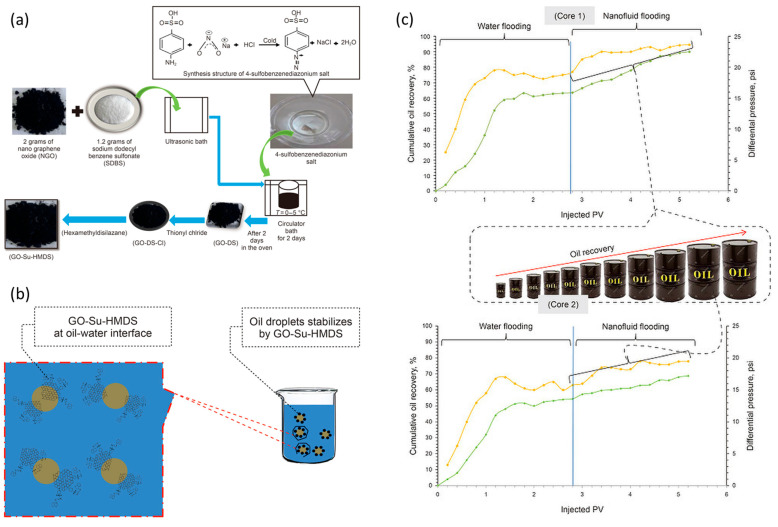
Effects of modified graphene oxide (GO) nanofluid on wettability and IFT changes. Adapted from Ref. [104]. (**a**) Step-by-step schematic view of NPs synthesis GO-Su-HMDS; (**b**) schematic representation of amphiphilic GO-Su-HMDS at the crude oil and water contact surface; (**c**) cumulative oil recovery and differential pressure in the process of water and synthesized GO-Su-HMDS nanofluid flooding at prepared concentrations of (Core 1) 400 and (Core 2) 500 ppm.

The influence of functionalized GO nanofluids on wettability alteration and interfacial tension reduction is illustrated in Figure 10. These results demonstrate how amphiphilic GO-based nanofluids can modify oil–water interfacial conditions and contribute to improved oil recovery during flooding experiments [104].

Taken together, these studies indicate that carbon-based nano-systems reduce heavy-oil flow resistance through a combination of structural disturbance of asphaltene aggregates and interfacial regulation of multiphase flow behavior. Unlike catalytic nano-systems used under thermal conditions, these mechanisms primarily operate through reversible physical interactions and are therefore particularly relevant to cold or low-energy heavy-oil production environments [87,89,92,103,104,105,108].

### 3.3. Mechanistic Comparison and Design Implications for Non-Metal Nano-Systems

The studies discussed in the preceding subsections indicate that a broad range of non-metal nanomaterials, particularly silica NPs and carbon-based materials such as graphene oxide (GO), have been investigated for heavy-oil viscosity reduction under non-thermal conditions [87,89,92,94,96,97,98,102,103,104]. In addition to these widely studied systems, emerging bio-derived nanomaterials such as cellulose nanocrystals (CNC) have also attracted attention because of their environmental compatibility and potential roles in nano-assisted emulsification processes [93]. However, their application in heavy-oil viscosity reduction remains limited. These studies suggest that the effectiveness of non-metal nano-systems is primarily governed by several common physicochemical pathways that influence heavy-oil mobility.

Despite differences in composition and morphology, silica- and carbon-based nanomaterials often affect heavy-oil flow through three closely related mechanisms: interfacial regulation, structural disturbance of asphaltene-rich networks, and dispersion-driven mobility enhancement [87,89]. The relative importance of these mechanisms depends on the NPs surface chemistry, formulation design, and reservoir conditions.

Interfacial regulation represents one of the most widely reported mechanisms, particularly for silica-based systems. Silica NPs can adsorb at mineral surfaces or oil–water interfaces, altering wettability and generating structural disjoining pressure that promotes detachment of oil films from rock surfaces [94,96,97]. In addition, partially hydrophobic or surface-modified silica NPs can stabilize oil-in-water emulsions through Pickering-type mechanisms, producing dispersed oil droplets within a continuous aqueous phase [63]. Under such conditions, apparent viscosity reduction is largely associated with the transition from continuous viscous oil flow to dispersed-flow regimes rather than intrinsic viscosity reduction in the crude oil [87,89].

Another important pathway involves the disruption of supramolecular association networks formed by asphaltenes and resins. Carbon-based nanomaterials, particularly GO, have been widely studied in this context. Due to their two-dimensional structure and large surface area, GO nanosheets can interact with aromatic components through π–π interactions and hydrogen bonding, enabling insertion into asphaltene nanoaggregates and weakening the connectivity of the aggregate network [102,103,104,106,107]. Such structural interference reduces the mechanical stability of viscoelastic networks responsible for high flow resistance, thereby improving heavy-oil mobility without requiring thermal upgrading.

In practical nano-assisted systems, these mechanisms rarely operate independently. NPs are often incorporated into multi-component formulations with surfactants or polymers, where viscosity reduction arises from combined effects of interfacial activity, dispersion stabilization, and partial disruption of asphaltene aggregates [88,97,98]. Surface functionalization of GO—such as polymer grafting, amphiphilic modification, or alkyl-chain functionalization—has been widely reported to improve dispersion stability and interfacial activity under reservoir conditions [103,109,110,111,112,113,114]. Such integrated formulations can simultaneously reduce interfacial tension, stabilize dispersed droplets, and modify wettability, thereby promoting dispersed-flow regimes and reducing flow resistance in porous media [63,87].

Quantitative comparisons further illustrate operational differences between representative nano-systems. Silica-based systems commonly operate at concentrations of approximately 100–5000 ppm and are frequently associated with Pickering-emulsion formation and wettability alteration mechanisms [97,100]. In contrast, GO-based systems often show effective performance at lower dosages, typically tens to several hundreds of ppm, where dispersion stability and surface functionalization play decisive roles [95,102,112,113,114]. Despite these differences in formulation and dosage, both systems primarily improve heavy-oil mobility through interfacial and structural regulation rather than irreversible compositional upgrading [87,89].

From a field-chemistry perspective, these observations suggest that the performance of nano-assisted viscosity-reduction systems depends not only on nanomaterial properties but also on dispersion stability in reservoir brines, controlled adsorption and retention in porous media, and compatibility with co-injected chemical agents [83,88,95,112,113,114]. These considerations indicate that viscosity reduction observed in laboratory systems must be evaluated together with transport behavior and operational constraints under reservoir conditions.

## 4. Reservoir-Scale Evaluation of Nano-Assisted Viscosity Reduction Systems

The preceding sections established that nano-assisted viscosity reduction in heavy oil can proceed through two fundamentally different pathways: catalytic upgrading under hydrothermal conditions and interfacial–structural regulation under non-thermal environments. However, under field conditions, the practical value of these mechanisms depends not only on their laboratory performance but also on whether they can operate within the thermal, hydraulic, and chemical constraints of real reservoirs. Studies on metal-based nano-catalysts confirm that their effectiveness remains strongly tied to sustained hydrothermal activation, meaning that their relevance decreases significantly once thermal stimulation is absent [115,116].

Cold-production environments, in contrast, magnify operational sensitivities that are less visible in thermal settings. When pressure gradients are limited and base mobility is poor, dispersion stability, NPs transport, and retention become decisive factors determining whether nanomaterials can reach and act within the reservoir [117,118]. Under these conditions, non-thermal mechanisms such as interfacial regulation, structural disjoining pressure, and dispersed-flow promotion may significantly influence heavy-oil mobility [119]. This distinction is especially relevant in reservoirs where cold production is selected because steam-based recovery is constrained by water demand, energy cost, or carbon-emission considerations. Life-cycle analyses of thermal recovery processes further emphasize that steam generation dominates both operational cost and environmental footprint [120].

Consequently, the practical performance of nano-assisted viscosity reduction must be evaluated from a reservoir-scale perspective that considers NPs stability, porous-media transport, and operational compatibility rather than relying solely on laboratory bottle tests. Therefore, the following sections examine the key reservoir-scale factors governing the applicability of nano-assisted systems.

### 4.1. NPs Transport and Stability in Porous Media

In cold-production reservoirs, the effectiveness of nano-assisted viscosity-reduction systems depends fundamentally on the ability of NPs to remain dispersed in reservoir brines and propagate through porous media. Reviews on nanofluid applications in enhanced oil recovery consistently identify dispersion stability, propagation distance, and retention control as the most influential determinants of field performance [121,122]. In this context, the central question is not only whether NPs can interact with heavy-oil components but also whether the formulation can remain stable and mobile under reservoir conditions [123].

A primary constraint arises from brine chemistry. Reservoir brines often exhibit high ionic strength and significant concentrations of divalent ions such as Ca^2+^ and Mg^2+^, which compress the electrical double layer surrounding NPs and accelerate aggregation processes [124]. Once aggregation occurs, the NPs’ surface area and interfacial activity decrease, while the effective particle size increases. This aggregation can severely compromise both interfacial functionality and porous-media transport.

Experimental studies investigating nanofluid stability under realistic reservoir conditions show that the stability window of NPs dispersions depends strongly on ionic composition, temperature, and surface chemistry [125,126,127,128]. Consequently, nanofluids evaluated only under deionized water conditions often fail to maintain stability when exposed to reservoir brines. Therefore, recent studies emphasize testing NPs systems under brines representative of actual field conditions [129,130,131].

This requirement applies equally to carbon-based nanomaterials such as graphene oxide. Although these systems can exhibit strong interfacial activity, their two-dimensional nanosheet structure makes them susceptible to aggregation when electrostatic stabilization is weakened by high salinity or multivalent ions [132,133]. Surface functionalization and steric stabilization are therefore frequently employed to maintain dispersion stability and preserve interfacial activity.

Beyond stability considerations, NPs transport and retention represent equally important constraints. During injection and propagation through porous media, NPs may be retained through adsorption on mineral surfaces, mechanical trapping in pore throats, and aggregation-induced deposition [134]. Retention processes are particularly critical in heavy-oil reservoirs where flow velocities are low and pressure gradients are limited.

Retention is closely linked to injectivity performance. Aggregation or deposition near the wellbore can increase flow resistance and reduce injectivity, thereby limiting the effective treatment radius [135]. For this reason, controlling particle size distribution and surface chemistry is essential for balancing interfacial functionality with acceptable transport behavior.

More recent studies also highlight that NPs’ retention does not always act purely as a negative factor. Moderate retention near oil–water or oil–rock interfaces may localize NPs at locations where interfacial regulation is most effective [136]. However, excessive retention can still dominate system behavior by limiting propagation distance and increasing injectivity risks [137].

Taken together, these observations demonstrate that reservoir-scale performance depends not only on NPs functionality but also on maintaining a balance between dispersion stability, transportability, and controlled retention within porous media.

### 4.2. Comparative Performance of Nano-Assisted Viscosity Reduction Systems

When examined from a reservoir-scale perspective, metal nano-catalysts and non-metal nano-systems operate within fundamentally different performance windows. Catalytic nano-systems rely primarily on reaction-driven upgrading mechanisms that require hydrothermal activation. Under cold-production conditions where such activation is absent, the contribution of catalytic upgrading to viscosity reduction is generally modest [115,116].

Non-metal nano-systems, by contrast, influence heavy-oil mobility primarily through interfacial and structural regulation mechanisms. Reviews of nanofluid EOR technologies consistently emphasize that these mechanisms include wettability alteration, interfacial film stabilization, dispersed-flow formation, and disturbance of asphaltene association networks [117,118,119].

The relative relevance of these mechanisms depends strongly on reservoir operating conditions. In reservoirs where thermal recovery is feasible, metal nano-catalysts can enhance catalytic aquathermolysis reactions and improve the efficiency of thermal processes [120]. However, when thermal recovery is restricted by environmental or economic constraints, non-metal nano-systems often become more attractive because they can operate under low-to-moderate temperature conditions.

Comparative evaluation of nano-systems must therefore consider not only peak viscosity reduction but also operational constraints such as dispersion stability, porous-media transport, and injectivity [121,122,123]. These constraints highlight that performance comparisons based solely on laboratory bottle tests may not accurately reflect field-scale applicability.

Table 3 summarizes the major operational dimensions influencing nano-system selection, including brine stability, transport and retention behavior, and injectivity constraints [134,135]. Building upon these considerations, Table 4 provides a systematic comparison of representative nano-assisted systems in terms of NPs type, concentration window, reservoir conditions, and dominant mechanisms.

Such comparative analysis illustrates that different nano-systems occupy distinct operational niches and should therefore be evaluated within reservoir-specific contexts rather than as universally applicable solutions.

### 4.3. Reservoir Applicability and System Selection Criteria

Because nano-assisted viscosity-reduction mechanisms differ substantially between thermal and non-thermal systems, selecting an appropriate nano-system requires careful consideration of reservoir conditions.

The first screening variable is whether hydrothermal activation is available. If the reservoir supports thermal recovery processes such as steam injections, metal nano-catalysts may provide additional benefits by enhancing catalytic upgrading reactions [115,116]. In such environments, NPs catalysts can work synergistically with thermal recovery to improve upgrading efficiency.

In contrast, when thermal recovery is constrained by water consumption, energy cost, or carbon-emission limitations, non-metal nano-systems become more relevant [117,118,119,120]. In these reservoirs, viscosity reduction is typically achieved through interfacial and structural mechanisms such as wettability alteration, dispersed-flow formation, and modification of asphaltene association networks.

Additional screening criteria include brine tolerance, transport behavior, and operational compatibility [121,122,123]. NPs formulations must remain stable under reservoir brines, propagate through porous media without excessive retention, and remain compatible with chemical flooding agents and produced-fluid handling systems.

From this perspective, the most effective nano-assisted systems are not necessarily those with the highest laboratory viscosity-reduction performance but rather those capable of maintaining stability, transportability, and functionality under realistic reservoir conditions.

### 4.4. Challenges in Scaling Nano-Assisted Systems from Laboratory to Field

Despite promising laboratory results, translating nano-assisted viscosity-reduction technologies to field-scale applications remains challenging.

One major limitation arises from the difference between laboratory experiments and reservoir conditions. Laboratory tests typically involve short core samples and simplified fluid compositions, whereas real reservoirs exhibit heterogeneity in permeability, mineralogy, and fluid chemistry [121,124]. These factors can significantly influence NPs’ stability and transport behavior.

Economic feasibility also represents an important consideration for large-scale implementation. Although some nanomaterials demonstrate strong laboratory performance at relatively low concentrations, the cost of large-scale synthesis and injection must be evaluated relative to the incremental oil recovery achieved [139].

Environmental and regulatory considerations are becoming increasingly important as well. Long-term NPs retention, potential remobilization, and interactions with reservoir geochemistry must be carefully evaluated to ensure safe deployment [138].

Taken together, these challenges indicate that the future development of nano-assisted viscosity reduction is shifting from simple NPs screening toward integrated nano-system design that considers reservoir conditions, formulation stability, and operational feasibility. Therefore, bridging the gap between laboratory studies and field-scale validation remains a central task for advancing nano-assisted technologies in heavy-oil recovery.

## 5. Reservoir-Oriented Design and Future Perspectives for Nano-Assisted Viscosity Reduction

Recent research on nano-assisted heavy-oil viscosity reduction has moved well beyond simple NPs screening. As discussed in the preceding sections, the practical performance of nano-assisted systems depends not only on whether NPs can reduce apparent viscosity or disturb asphaltene-rich structures under laboratory conditions, but also on whether these effects can be sustained under realistic reservoir constraints. In this sense, the current development trajectory is shifting from isolated material optimization toward system-oriented formulation design, in which nanomaterials are treated as functional modules embedded within engineered flooding systems. In cold or low-to-moderate temperature environments, such systems are increasingly organized around three complementary components: (i) a structural or interfacial regulator, such as surface-engineered silica or graphene-derived materials, (ii) an interfacial package based on surfactants or amphiphiles to control interfacial tension and film properties, and (iii) a stability or transport stabilizer, often polymeric in nature, to maintain dispersion integrity during injection and propagation [103,105,109,110,140]. A consistent trend emerging from recent reviews is that the field is placing greater emphasis on operational descriptors—such as stability under realistic brines, injectivity and retention risk, and propagation distance—rather than on viscosity reduction in bottle tests alone [121,141].

### 5.1. Bridging Laboratory Mechanisms and Reservoir Applications

A central challenge for nano-assisted viscosity reduction is the persistent gap between laboratory mechanisms and reservoir-scale applications. Most available studies still rely on bottle tests, rheological measurements, short core floods, or simplified brine systems to evaluate NPs’ performance. These approaches remain essential for identifying interfacial and structural mechanisms, but they do not fully reproduce the coupled constraints that determine performance in the field [141]. In actual reservoirs, NPs formulations must remain stable in high-salinity brines, propagate through porous media with acceptable retention, interact effectively with heavy-oil microstructures and interfaces, and do so without causing excessive injectivity decline or produced-fluid handling problems [121,124].

For cold heavy-oil production, the practical gate is therefore not whether a nano-system can merely “interact with asphaltenes”, but whether the formulation can remain dispersed under reservoir brine conditions, move through porous media with controllable retention, and stay compatible with the field chemical slate, including surfactants, polymers, and other additives [64,83,122,123]. Brine tolerance is often the first failure mode. High ionic strength and divalent ions compress electrostatic repulsion, accelerate aggregation, and reduce effective interfacial activity [64,89,124]. In this context, salinity should not be treated as a single scalar value but as a mechanistic variable: ion identity, hardness, and competitive adsorption can all influence colloidal stability and retention behavior in different ways, even at similar total dissolved solids [142].

This lab-to-field translation problem is especially evident for silica-based and graphene-derived systems. For silica nanofluids, experimental studies under reservoir-representative salinity and temperature conditions show that stable operating windows are formulation-specific and cannot be inferred reliably from deionized-water screening alone [125]. Reviews on NPs surface engineering similarly emphasize that post-synthesis functionalization—through polymer, surfactant, salinization, or related modification strategies—is now central to extending nanofluid applicability under harsh brines while preserving interfacial function [143]. Thus, bridging laboratory mechanisms to field application requires a more integrated experimental philosophy: mechanism identification must be coupled with stability, transport, and formulation testing under realistic reservoir conditions.

### 5.2. Design Principles for Nano-Assisted Viscosity Reduction Systems

The literature reviewed in this work suggests that successful nano-assisted viscosity reduction should be approached as a system-level formulation challenge rather than as a search for a single “best” nanomaterial [64,121,124]. From this perspective, several design principles can be extracted.

First, dispersion stability under reservoir brines should be treated as a primary requirement rather than a secondary property. A nano-system that loses colloidal stability under salinity, divalent ions, or shear cannot sustain transport or interfacial activity at the reservoir scale. This requirement applies to both silica-based and graphene-derived systems, although the stabilization strategies may differ. For non-metallic systems intended for cold production, stability should always be validated in representative brines and over practical temperature ranges.

Second, transport and retention must be balanced rather than minimized blindly. Some adsorption and retention may be beneficial if they localize NPs near oil–water or oil–rock interfaces where interfacial regulation is needed. However, excessive retention increases propagation loss and injectivity risk. The practical objective is therefore not zero retention, but controllable and reservoir-compatible retention.

Third, formulation integration is generally more important than standalone NPs performance. Increasingly, the literature points toward combining nanomaterials with conventional chemical flooding agents such as surfactants and polymers to generate synergistic effects on interfacial tension reduction, wettability alteration, dispersed-flow stabilization, and bulk mobility control [140]. This trend is also consistent with recent work on environmentally compatible and green nanocomposite systems, which indicates that future formulations are likely to rely more on hybrid design than on single-component nanofluids [144].

Fourth, performance should be judged by delivered effect rather than nominal material cost or bottle-test viscosity reduction alone. A low-cost NPs with poor brine tolerance or severe retention may be less useful than a more expensive but lower-dosage, better-transporting formulation. Thus, formulation design should consider cost per effective reservoir response, not simply unit material cost.

Taken together, these principles imply that the most credible cold-production nano-systems are those engineered as robust formulations that remain dispersed under brine conditions, propagate with acceptable retention, improve mobility through interfacial and structural regulation, and do so without creating disproportionate penalties in injectivity or produced-fluid separation [64,83,124,134,135].

### 5.3. Future Research Directions

Future research should move in three coordinated directions.

First, more work is needed on reservoir-representative validation. This includes long-core or heterogeneous-core flooding, dynamic stability testing under field brines, and a more systematic evaluation of NPs transport, retention, and injectivity behavior under mobility-limited conditions. Such studies are essential if the field is to move beyond descriptive laboratory promise toward predictive reservoir applicability.

Second, research should continue advancing integrated and surface-engineered nano-systems. This includes more robust interfacial packages, polymer-assisted stabilization strategies, and formulation architectures capable of withstanding salinity, shear, and porous-media transport simultaneously. In parallel, environmentally compatible and green nano-systems deserve increasing attention, provided that their stability and transport performance can be demonstrated under realistic reservoir conditions [144].

Third, future evaluations of nano-assisted viscosity reduction should include economic, environmental, and operational metrics more explicitly. Large-scale deployment will depend not only on technical performance but also on supply-chain maturity, formulation scalability, and environmental accountability [145]. Recent methane-emission analyses of heavy-oil production systems, including CHOPS-related operations, further highlight that future recovery technologies must increasingly be evaluated within broader low-carbon and emissions-management frameworks [145,146]. In this sense, economic feasibility, scalability, and environmental compatibility should be treated as core design constraints rather than afterthoughts.

Overall, the future of nano-assisted viscosity reduction lies less in discovering entirely new nanomaterials than in developing reservoir-specific, operationally robust nano-systems. Progress will depend on integrating mechanistic understanding, formulation engineering, porous-media transport control, and field-oriented validation into a unified design framework. Only through such a transition can nano-assisted viscosity reduction evolve from a promising laboratory concept into a reliable and scalable technology for heavy-oil recovery.

## 6. Summary and Outlook

This review examines recent advances in nano-assisted viscosity reduction for heavy-oil recovery, with particular emphasis on the transition from thermally driven catalytic upgrading toward interfacial and structural mobility-control strategies applicable under cold-production conditions. By organizing the literature according to reservoir-operational environments rather than simply NPs categories, the review distinguishes between metal-based nano-systems that rely on catalytic aquathermolysis under hydrothermal conditions and non-metal nano-systems that regulate interfacial properties and heavy-oil microstructure at low or moderate temperatures. Integrating mechanistic understanding with reservoir-scale considerations provides a more practical perspective for evaluating nano-assisted viscosity-reduction technologies and clarifies the conditions under which different nano-systems may contribute to improved heavy-oil mobility. The main conclusions of this review can be summarized as follows:

(1) Mechanism-level conclusions. Nano-assisted viscosity reduction in heavy oil generally proceeds through two distinct mechanistic pathways. Metal-based NPs mainly catalyze aquathermolysis reactions under hydrothermal conditions, promoting bond cleavage and hydrogen-transfer reactions that partially upgrade heavy fractions and lead to persistent viscosity reduction during thermal recovery. In contrast, non-metal nanomaterials—particularly silica-based and graphene-derived systems—primarily operate through interfacial and structural regulation mechanisms, including wettability alteration, stabilization of oil–water interfacial films, modification of asphaltene aggregation behavior, and the formation of dispersed-flow regimes that reduce apparent flow resistance in multiphase systems.
(2) Reservoir-scale insights. From a reservoir-engineering perspective, the effectiveness of nano-assisted viscosity reduction depends not only on NPs functionality but also on operational factors such as dispersion stability under reservoir brines, NPs transport and retention in porous media, and compatibility with injection processes. These factors determine whether laboratory-scale viscosity-reduction mechanisms can be sustained during field operations and therefore represent key constraints for practical implementation.
(3) Comparative implications. Comparative analysis of nano-assisted systems indicates that metal-based nano-catalysts are most effective when integrated with thermal recovery processes, where sustained hydrothermal conditions activate catalytic upgrading reactions. In contrast, non-metal nano-systems are more suitable for cold or low-temperature production environments where mobility improvement is achieved mainly through interfacial regulation and structural disturbance rather than irreversible chemical upgrading.
(4) Design framework for nano-assisted viscosity reduction. Successful field deployment of nano-assisted viscosity-reduction technologies requires reservoir-oriented formulation design rather than the selection of individual nanomaterials alone. Effective nano-systems should combine stable dispersion under reservoir brines, controlled transport and retention behavior in porous media, and sufficient interfacial activity to modify wettability or stabilize dispersed-flow regimes. In many cases, integrating nanomaterials with conventional chemical flooding agents such as surfactants or polymers may provide more robust and operationally feasible solutions for improving heavy-oil mobility.

Looking forward, the development of nano-assisted viscosity-reduction technologies is likely to move toward integrated nano-systems designed for specific reservoir environments rather than toward the discovery of individual nanomaterials alone. In particular, improving dispersion stability under reservoir brines, controlling NPs transport in porous media, and integrating nanomaterials with existing chemical flooding systems will be essential for translating laboratory mechanisms into practical field applications. Such reservoir-oriented nano-system design may provide new opportunities for improving heavy-oil mobility under both thermal and cold-production conditions.

## Figures and Tables

**Table 1:** The summary of information from review papers on the viscosity reduction in heavy oil.

Reference	Main Theme/Key Findings (Condensed)	Cited Literature Year Range	Main Materials/Nano-Systems	Limitations/Constraints
Ke, H. et al. [58]	General review of nanomaterials for viscosity reduction, covering mechanisms like catalytic aqua thermolysis, magnetic effects, and functionalization.	2010–2020	SiO_2_, metal oxides (Fe_3_O_4_, Al_2_O_3_, NiO), CNTs, nanocomposites.	NP aggregation, stability issues, and limited reservoir-scale evidence.
Kharisov, B. I. et al. [59]	Focuses on materials for the removal/adsorption of heavy oil components (asphaltenes, resins), a key cold-reduction mechanism.	2005–2016	Nanostructured iron oxides, nickel-containing NPs, and graphene oxide (GO).	Strong dependence on surface chemistry; unpredictable adsorption in real reservoirs.
Li, X. et al. [4]	Reviews various new viscosity reduction technologies, including non-chemical/non-thermal physical methods (e.g., ultrasonic and magnetic techniques).	2015–2021	Covers chemical and physical technologies.	The scope is very broad, extending to non-nanomaterial methods.
Hashemi, R. et al. [60]	Overview of NPs for in situ upgrading and recovery enhancement. Reviews catalytic upgrading and EOR mechanisms.	2008–2014	Metal NPs (Ni, Mo), supported oxides (SiO_2_, Al_2_O_3_, CeO_2_).	Challenges facing NP applications, including reduction in costs, transport, and stability.
Gao, C. et al. [45]	Reviews chemical reducers (surfactants, polymers, catalytic) to avoid high energy consumption and carbon emission from thermal recovery. Focuses on application strategies and green circulation.	2000–2024	Primarily chemical agents. Mentions NPs as surfactant additives.	High energy consumption/carbon emissions. Need for green development and cost control of reducers.
Bohorquez, L. C. et al. [61]	Review of nanotechnology for viscosity reduction, discussing physicochemical properties and mechanisms like adsorption, IFT reduction, and deasphalting.	1990s−2017	SiO_2_ (functionalized), carbon materials, metal oxides.	High viscosity negatively impacts production/recovery/transport costs.
Zhou, W. et al. [14]	Comprehensive review covering NP applications in nanofluid flooding and as hybrids with traditional methods (thermal, chemical, gas).	2018–2023	SiO_2_, Fe_3_O_4_, Al_2_O_3_, CNTs, graphene, nanocellulose.	High costs, significant energy/water consumption, and GHG emissions of traditional methods.
Medina, O. E. et al. [62]	Review of nanotechnology in thermal EOR (steam flooding, ISC, EM heating); highlights catalytic thermal cracking and NP–asphaltene interactions.	2000–2019	Catalytic metal oxides (e.g., NiO–PdO functionalized SiO_2_).	Thermal EOR is energy-intensive; uncertain NP performance under harsh conditions
Arab, D.; Kantzas, A.; Bryant, S. L. [63]	Reviews NPs-stabilized oil-in-water emulsions for heavy-oil mobility improvement; highlights interfacial adsorption and wettability effects; viscosity reduction mainly arises from dispersed-flow behavior.	Up to 2017	Silica NPs; surface-modified NPs; emulsion-based systems.	Strong dependence on surface chemistry and emulsion stability; separation challenges.
Kandiel, Y. E. et al. [64]	Reviews a broad range of NPs for EOR, including silica, metal oxides, and carbon-based materials; highlights emerging trends in environmentally friendly nanomaterials.	Up to 2024	Silica, metal oxides, carbon-based nanomaterials, and emerging nanomaterials.	Limited discussion on specific material classes for heavy-oil viscosity reduction; field-scale validation is still limited.

**Table 2:** Representative catalytic aquathermolysis studies using metal-based nano-catalysts for heavy-oil viscosity reduction.

Nano-Catalysts	Temperature (°C)	Concentration (ppm)	Viscosity Reduction	Limitations/Constraints	Mechanism	Reference
Fe_3_O_4_ NPs	240–260	1000–3000	50–75%	Asphaltene ↓, Saturates ↑	catalytic aquathermolysis and hydrogen transfer	[77]
NiO NPs	260–300	1000–2000	60–85%	Resin ↓, Light fractions ↑	C–S bond cleavage and hydrogen redistribution	[76]
Ni–Fe bimetallic NPs	280–300	1000	70–90%	Asphaltene ↓	synergistic catalytic upgrading	[72]
Fe_2_O_3_ NPs	220–260	500–1000	40–65%	Moderate reduction in heavy fractions	aquathermolysis catalysis	[70,71]
CeO_2_ supported catalysts	200–240	500	30–55%	Limited SARA change	catalytic activation of heavy fractions	[65]

**Table 3:** Practical screening summary of nano-systems for heavy-oil viscosity management (cold-production perspective).

Screening Dimension	Metal Nano-Catalysts (Thermal-Oriented)	Non-Metal Nano-Systems (Cold-Oriented)	Representative Refs.
Dominant lever	Reaction-driven upgrading (aqua thermolysis; bond cleavage; H-transfer)	Interfacial regulation + structural control (wettability, interfacial films, dispersion/emulsification, network weakening)	[70,88,99,116,118]
Temperature window	Strongest under steam/hot-water conditions	Effective at low-to-moderate temperatures if stable and transportable	[70,116,118,121]
Brine stability as a gating factor	Stabilization required; aggregation undermines delivery	Often, the primary design challenge is surface modification/formulation critical	[64,122,124,125,126,127,128,129,130,131]
Transport/retention and injectivity (often the limiting factor for field deployment)	Retention risk must be managed; near-wellbore impacts can dominate	Same constraint: aggregation-driven size increase is particularly penalizing	[83,122,124,134,135]
Role of adsorption	Can aid contact, but excessive adsorption increases retention	Prefer partially reversible adsorption as an enabler, not the main driver	[83,88,98,125]
Surface-facility implications	Typically, not emulsification-driven	Dispersion/emulsification can aid mobility, but must remain manageable	[64,88,99]
Scalability and cost	Tied to thermal-input economics and footprint	More scalable if low-cost materials + robust formulation are achieved	[64,120,138]
Environmental/monitoring burden	Needs fate/retention assessment	Increased scrutiny for functionalized carbon nanomaterials	[124,138,139]

**Table 4:** Field-operational summary of nano-assisted viscosity-reduction systems.

Nano-System (Typical)	Concentration Window	Initial Oil Viscosity (Typical)	Temperature	Brine Condition Sensitivity	Dominant Mechanism	Key Refs.
Silica (Pickering-type)	100–5000 ppm	~10^3^–10^6^ mPa·s	Low–moderate	needs dispersion under salinity; divalent ions matter	Interfacial-film strengthening; dispersed-flow regime; apparent-viscosity reduction	[63,97,100]
GO/functionalized GO	~10–1000 ppm	~10^3^–10^6^ mPa·s	Low–moderate	salinity/pH/divalent-ion sensitivity; retention risk	Interfacial regulation + weakening of asphaltene-rich associations; mobility control	[92,103,105,108]
Nano + chemical formulation (emulsification/dispersion)	Depends on the recipe	~10^3^–10^6^ mPa·s	Low–moderate	brine tolerance and separation constraints	Formulation-stabilized dispersion/emulsification for mobility improvement	[103,105,114,124,134]
Metal/metal-oxide (thermal-bound)	case-specific	often higher-viscosity fractions	Hydrothermal	transport/retention under steam	Catalytic aqua thermolysis/partial upgrading (thermal-bound)	[65,70,72,76]

## Data Availability

The original contributions presented in this study are included in the article. Further inquiries can be directed to the corresponding authors.
